# Civil Malpractice

**Published:** 1873-02

**Authors:** 


### *Civil Malpractice.*

In the present No. of the JOURNAL is commenced an elaborate article on this subject which we cordially commend to the careful perusal of our readers. Such of our legal friends as have looked it over in advance sheets speak highly of its merits, and we are sure our professional brethren will cordially indorse their opinion. It covers a space which the valuable work of Elwell does not embrace.

